# Hyperthermia Influences the Secretion Signature of Tumor Cells and Affects Endothelial Cell Sprouting

**DOI:** 10.3390/biomedicines11082256

**Published:** 2023-08-12

**Authors:** Wisdom O. Maduabuchi, Felista L. Tansi, Regine Heller, Ingrid Hilger

**Affiliations:** 1 Department of Experimental Radiology, Institute of Diagnostic and Interventional Radiology, Jena University Hospital—Friedrich Schiller University Jena, Am Klinikum 1, D-07747 Jena, Germany; wisdom.maduabuchi@med.uni-jena.de (W.O.M.); felista.tansi@med.uni-jena.de (F.L.T.); 2 Institute of Molecular Cell Biology, Center for Molecular Biomedicine (CMB), Hans-Knöll-Str. 2, D-07745 Jena, Germany; regine.heller@med.uni-jena.de

**Keywords:** hyperthermia, pancreatic ductal adenocarcinoma (PDAC), endothelial cells (EC), sprouting angiogenesis, vascular endothelial growth factor (VEGF), extracellular signal-regulated kinase (ERK)

## Abstract

Tumors are a highly heterogeneous mass of tissue showing distinct therapy responses. In particular, the therapeutic outcome of tumor hyperthermia treatments has been inconsistent, presumably due to tumor versus endothelial cell cross-talks related to the treatment temperature and the tumor tissue environment. Here, we investigated the impact of the average or strong hyperthermic treatment (43 °C or 47 °C for 1 h) of the human pancreatic adenocarcinoma cell line (PANC-1 and BxPC-3) on endothelial cells (HUVECs) under post-treatment normoxic or hypoxic conditions. Immediately after the hyperthermia treatment, the distinct repression of secreted pro-angiogenic factors (e.g., VEGF, PDGF-AA, PDGF-BB, M-CSF), intracellular HIF-1α and the enhanced phosphorylation of ERK1/2 in tumor cells were detectable (particularly for strong hyperthermia, 2D cell monolayers). Notably, there was a significant increase in endothelial sprouting when 3D self-organized pancreatic cancer cells were treated with strong hyperthermia and the post-treatment conditions were hypoxic. Interestingly, for the used treatment temperatures, the intracellular HIF-1α accumulation in tumor cells seems to play a role in MAPK/ERK activation and mediator secretion (e.g., VEGF, PDGF-AA, Angiopoietin-2), as shown by inhibition experiments. Taken together, the hyperthermia of pancreatic adenocarcinoma cells in vitro impacts endothelial cells under defined environmental conditions (cell-to-cell contact, oxygen status, treatment temperature), whereby HIF-1α and VEGF secretion play a role in a complex context. Our observations could be exploited for the hyperthermic treatment of pancreatic cancer in the future.

## 1. Introduction

Tumors are a highly heterogeneous mass of tissue consisting of diverse cell populations with different physiological features and distinct therapy responses [1]. The heterogeneity of tumors is in part responsible for the evolution of resistant phenotypes promoted by selective therapy stress [1]. Therapy resistance is also induced by the structural heterogeneity of the tumor microenvironment. Moreover, alterations in the blood flow and oxygen concentration may modify the delivery and efficacy of therapeutic agents [2,3]. Finally, the tumor cell’s interaction with its adjacent stromal cells is also responsible for therapy resistance, as these interactions may cause abnormal responses that allow cancer cells to survive otherwise fatal therapies [2]. 

Hyperthermia involves the use of specific high temperatures in cancer treatment [4,5]. It comprises the application of temperatures over 37 °C at the site of the tumor to incite pathophysiological changes that will eventually lead to cell death [6]. Several research studies have shown that temperatures between 41 to 47 °C can induce cell death and the activation of several pathways in cancer cells [4,7]. The effectiveness of hyperthermia depends not only on the temperature achieved, but also on the duration of exposure [7]. The most commonly used temperatures of 43 °C and 45 °C (for 60 to 90 min) were found to induce cell death by activating all apoptotic pathways [8]. Interestingly, hyperthermia at 47 °C seems to have a greater effect on cell death than hyperthermia at 43 °C [9]. Nevertheless, limited knowledge is available regarding the effect of different temperature doses (e.g., 43 and 47 °C for 60 min) on angiogenesis. 

Basically, angiogenesis is a highly regulated process of vessel formation performed by endothelial cells (ECs) in response to chemical or mechanical signals induced by pathological conditions (such as cancer), tissue damage or nutrient and oxygen demand [10,11,12]. Sprouting angiogenesis occurs when ECs are activated. The activated ECs, called tip cells, migrate, degrading the extracellular matrix as they advance. Trailing behind are the stalk cells, which proliferate and follow the tip cells, elongating the sprout [10,11]. Interestingly, a different impact of hyperthermia on tumor angiogenesis has been reported so far. For example, high-dose hyperthermia at 46 °C for 1 h was observed to induce angiogenesis in breast adenocarcinoma (MDA-MB-231) in vivo [13]. Whole-body hyperthermia in rats (42 °C for 20 min) led to a significant increase in VEGF expression and neovascularization in cardiac tissues [14]. In contrast, hyperthermia (43 °C or 47 °C for 1 h) was also shown to favor anti-angiogenic processes, such as the suppressed production and gene expression of VEGF-A_165_ in human fibrosarcoma HT-1080 cells [15]. Further, reports revealed complete hyperthermia-mediated arrest during EC differentiation into vascular structures [16]. In this context, average hyperthermia (43 °C, 1 h) effectively inhibited EC morphology [17] and sprouting, even upon induction by VEGF-A_165_ (spheroid assay). However, hyperthermia did not alter pre-existing capillary structures, suggesting the selective inhibition of EC differentiation into sprouts [16]. Tumor neo-angiogenesis is known to be particularly triggered in the presence of hypoxic conditions [18,19,20]. One important player is the hypoxia-inducible factor 1 (HIF-1), which promotes endothelial sprouting via the regulation of various pro-angiogenic growth factors, such as the vascular endothelial growth factor A (VEGF-A) [21]. 

Under hypoxic conditions, HIF-1α generally plays a key role in regulating various cellular processes in tumor cells. HIF-1α is a transcription factor that regulates the expression of numerous genes involved in angiogenesis, glucose metabolism, cell proliferation, and apoptosis [22]. The upregulation of HIF-1α under hypoxia is a major driver of tumor progression and contributes to tumor growth, metastasis, and resistance to therapy [20]. Increased HIF-1α expression has been reported in hyperthermia-treated lung cancer cell lines (NCI-H1650 and NCI-H446), with higher expression observed at higher temperatures. Furthermore, extracellular signal-regulated kinase (ERK) signaling was found to regulate HIF-1α expression and tumor growth in hyperthermic non-small-cell lung cancer both in vitro and in vivo [21]. Several reports show that the ERK/MAPK pathway promotes angiogenesis by activating transcription factors that increase VEGF transcription and gene expression in tumor cells [23,24]. 

Despite our current knowledge, it remains unclear how hyperthermia modifies the cross-talk between pancreatic adenocarcinoma cells and endothelial cells. In this work, we verified how hyperthermia at different temperature doses (43 °C or 47 °C for 1 h) affects (1) the paracrine (pro-angiogenic) mediator secretion, intracellular HIF-1α accumulation and the ERK phosphorylation of pancreatic adenocarcinoma cells, and (2) sprouting angiogenesis in endothelial spheroids with dependence on the post-treatment oxygen environment and the cell-to-cell contact status of the pancreatic tumor cells (cell monolayers (2D) or spheroids (3D)). To understand how hyperthermia affects the role of hypoxia in the down-stream secretion of growth factors in pancreatic tumor cells, HIF-1α inhibition studies were performed. A better understanding of the mentioned interactions will help to improve the hyperthermic treatment of pancreatic adenocarcinoma as adjuvant to chemotherapy.

## 2. Materials and Methods

### 2.1. Cell Lines, Media and Cell Culture 

**Figure 1:**
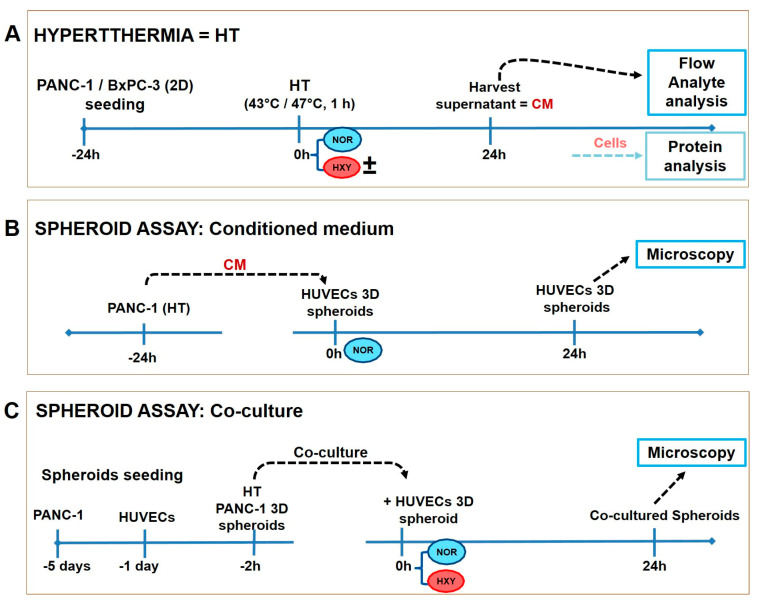
Schematic representation of the experimental workflow. Steps involved in investigating the effects of hyperthermia on the production of growth factors by pancreatic adenocarcinoma cells. Hypoxia was accomplished by culturing cells in a hypoxia chamber with 1% O_2_. (**A**) Cells were exposed to hot air hyperthermia at 43 or 47 °C for 1 h (average and strong hyperthermia, respectively), then further cultured at normoxia (37 °C, 5% CO_2_, 20% O_2_) or hypoxia (1% O_2_) with or without the inhibitor CAY10585(±) for 24 h. (**B**) Workflow for investigating the cross-talk between hyperthermia-treated PANC-1 cells on human endothelial spheroids (HUVECs): a conditioned medium (CM) from hyperthermia-treated PANC-1 cells was used to treat HUVEC spheroids. (**C**) Workflow for validating the effects of hyperthermia-treated PANC-1 spheroids on the sprouting potential of HUVEC spheroids under co-culture conditions of normoxia or hypoxia. NOR = Normoxia; HXY= Hypoxia; ± = with/without HIF-1α Inhibitor; CM = conditioned medium; HT = Hyperthermia (1 h, 43 or 47 °C).

We used the human pancreatic adenocarcinoma cell lines (PANC-1 and BxPC-3, both from ATCC) as a model of tumor cells with different levels of resistance to chemotherapy (e.g., gemcitabine, [25]). PANC-1 and BXPC-3 cells were cultured in DMEM and RPMI 1640 medium, respectively, supplemented with 10% (*v*/*v*) fetal bovine serum (Life Technologies GmbH, Darmstadt, Germany) and 1% penicillin/streptomycin (Life Technologies Corporation, Grand Island, NY, USA). Human umbilical vein endothelial cells (HUVEC) were employed as a model for primary endothelial cells. These cells were cultured in medium 199 (Lonza Inc., Walkersville, MD, USA) supplemented with 17.5% (*v*/*v*) fetal bovine serum (Sigma, Taufkirchen, Germany), 2.5% (*v*/*v*) human serum (HS) (Sigma, Taufkirchen, Germany), 7.5 µg/mL endothelial growth supplement (Biomedical Technologies Inc., Madrid, Spain), 7.5 U/mL heparin (Sigma, Taufkirchen, Germany), 680 µM glutamine (Lonza Inc., Walkersville, MD, USA) and 1% (*v*/*v*) penicillin/streptomycin (Lonza Inc., Walkersville, MD, USA). Cell culture was performed under standard conditions in a humidified atmosphere (37 °C, 20% O_2_, 5% (*v*/*v*) CO_2_). To understand the cross-talk between hyperthermia-treated tumor cells and endothelial cells (see below), we analyzed the conditioned medium from hyperthermia-treated PANC-1 or BxPC-3 cells. For these studies, we used 2D cell monolayers, since they show reproducible lateral cell-to-cell interactions, and a controlled oxygen and nutritional environment. To assess the impact of hyperthermia on endothelial cell sprouting, we used PANC-1 spheroids treated or not with hyperthermia for co-cultivation with HUVEC spheroids (see below). For these experiments, we used a 3D cell culture spheroid model of PANC-1 to mirror its effects in solid tumors, particularly the ability of cells to self-organize and produce their own extracellular matrix and to undergo cell-to-cell and cell-to-matrix interactions [26]. We further used HUVEC spheroids to model intratumoral endothelial cell “nests”, which are commonly present in tumors. All these experiments were performed under normoxia or hypoxia conditions. The schematic diagram illustrates the implemented workflow (Figure 1).

### 2.2. Preparation of PANC-1 and HUVEC (3D) Spheroid Cultures 

To obtain the PANC-1 spheroids, the cells were cultured in a 96-well flat-bottomed plate pre-coated with 50 µL of 2% (*w*/*v*) low-melting point agarose (Carl Roth GmbH + Co. KG, Karlsruhe, Germany) in medium. Briefly, 200 µL of culture medium containing 1500 PANC-1 cells was pipetted into each well of the pre-coated plates and cultured under standard conditions for five days. Every two days, 100 µL of the culture medium was replaced with fresh pre-warmed medium. For the HUVEC spheroids production, 1.2% (*w*/*v*) methylcellulose (Sigma, Taufkirchen, Germany) was added at a ratio of 5:1 to complete endothelial cell growth medium. Then, 100 µL of the methylcellulose cell suspension containing 3000 HUVECs was pipetted into each well of a round-bottomed 96-well plate and cultured for 24 h at standard culture conditions [27]. 

### 2.3. Hot Air Hyperthermia and Induction or Inhibition of Post-Therapeutic Hypoxia Effects 

The mentioned pancreatic carcinoma cell culture models (2D or 3D culture model, seeded in well plates) were subjected to hyperthermia by placing them in an incubator (Thermo Scientific™ HERAcell 150i, Langenselbold, Germany) for 60 min, which was previously set to temperatures of 43 °C (average hyperthermia) or 47 °C (strong hyperthermia). Then, 43 °C was used to investigate the cytotoxic effects of mild temperature, while 47 °C was used to investigate the acute cytotoxic effects of ablative temperatures [7]. The temperature was monitored using the FoTempMK-19 fiber-optic temperature sensor (Optocon AG, Dresden, Germany) positioned in the culture medium during the hyperthermic treatment. Typical temperature curves during hyperthermia treatment are shown in supplementary Figure S1. After hyperthermia, further culturing under standard conditions at 37 °C for 24 h or 48 h was performed. Post-treatment hypoxia was achieved by incubating PANC-1 monolayers or 3D spheroids in a designated incubator (HERAcell 150i, Germany) set to 1% (*v*/*v*) O_2_ and 37 °C for 24 h or 48 h. Only sub-confluent cultures were used for the experiments. HIF-1α inhibition was performed to understand how hyperthermia affects hypoxia’s role in the down-stream secretion of growth factors in pancreatic tumor cells. For the inhibition of HIF-1α in pancreatic tumor cells after hyperthermia, PANC-1 and BxPC-3 cell lines were treated with 10 μM of CAY10585 and then post-incubated in hypoxia (1% (*v*/*v*) O_2_, 37 °C) for 24 h. For these experiments, we used tumor cell monolayers, since a controlled nutritional environment was feasible with this model (see above). The schematic diagram illustrates the workflow implemented (Figure 1).

### 2.4. Cell Viability Assays

Following the hyperthermia treatment of PANC-1 cell monolayers, cell viability was determined using (1) the Alamarblue^®^ assay at 0, 24 and 48 h post-hyperthermia; it involved the use of resazurin-based solutions (excitation: 530–560 nm/emission: 590 nm) that serve as indicators of the metabolic activity of cells (for details see [28]), (2) counting cells via the CASY counter impedance spectroscopy (OMNI Life science); or (3) assessing the colony survival of cells at 15 days post-hyperthermia [29]. The levels of treated cells were related to the non-treated controls. All experiments involved 3 biological replicates with quadruple to sextuple samplings for each replicate. 

### 2.5. Flow Analyte Analysis of Growth Factors Secreted by the Pancreatic Tumor Cell lines 

The LEGENDplex^TM^ multi-analyte flow assay kits (Biolegend, San Diego, CA, USA) for the human growth factor panel (13-plex) and the human inflammation panel (13-plex) were used to investigate the growth factors secreted by PANC-1 and BxPC-3 cells into the medium under hyperthermia stress conditions. The LEGENDplex^TM^ is a bead-based immunoassay, which uses fluorescence-encoded beads that can be quantified via flow analyte analysis. Briefly, following hot air hyperthermia treatment and medium change, pancreatic cancer cells were cultured at 37 °C for a further 24 h under conditions of normoxia or hypoxia. After 24 h, the supernatant was harvested, and a multi-analyte flow assay was performed (Figure 1A). All experimental procedures were performed according to the manufacturer’s instructions, whereby 5 µL of culture supernatant was used per test. For flow analyte analysis, all samples were duplicated in a 96-well plate and growth-factor-bound fluorescent beads were analyzed using a BD Accuri flow cytometer (BD, Heidelberg, Germany). The LEGENDplex™ data analysis software v8.0 (BioLegend, San Diego, CA, USA) was used to evaluate the data. The concentration of secreted growth factors (pg/mL) was normalized to the cell count.

### 2.6. Treatment of HUVECs Spheroids with Conditioned Medium (CM) from Hyperthermic-PANC-1 Cells

To assess whether pancreatic tumor cells are able to induce sprouting in endothelial cell “nests” (see above), a conditioned medium from the hyperthermia-treated PANC-1 cell monolayers (2D, controlled nutrient and oxygen environment) was prepared and added to HUVEC spheroids. To this end, the condition medium was prepared as follows: PANC-1 cells were exposed to hyperthermia in an incubator at 43 or 47 °C for 1 h. To enable the adequate secretion of mediators, PANC-1 cells were further cultured for 24 h at standard conditions, before harvesting the conditioned culture medium. Proteinase inhibitor cocktail (Sigma, Taufkirchen, Germany) was added to the harvested conditioned medium. In parallel, HUVEC spheroids were prepared and cultured under standard conditions for 24 h. Afterwards, the HUVECs spheroids were embedded in fibrin gels (Merck KGaA, Darmstadt, Germany) in 24-well plates and the respective PANC-1 hyperthermia conditioned media (HT-CM) was immediately added to the HUVEC spheroids. For the negative and positive controls of HUVEC sprouting, media from untreated PANC-1 cultured at 37 °C and VEGF_165_ (R&D Systems, Inc., Minneapolis, MN, USA) at 10 ng/mL were added to the spheroids. The spheroids were incubated under standard conditions for a further 24 h, then fixed and analyzed for sprout formation (Figure 1B). 

### 2.7. Co-Culture of Hyperthermia-Treated PANC-1 3D Spheroids and HUVECs 3D Spheroids

To investigate the cross-talk between pancreatic tumor cells intratumoral endothelial cell “nests” (3D, high degree of cell-to-cell and cell-to-matrix interactions), we used a PANC-1 and HUVEC spheroid co-culture system. We used PANC-1 cells, since this pancreatic tumor cell line shows several mutations associated with invasiveness (mutation of the K-ras, p53, p16, SMAD4 genes, etc. [30]). To this end, PANC-1 spheroids prepared as described above were subjected to hyperthermia at 43 or 47 °C for 1 h. Immediately after hyperthermia, the PANC-1 spheroids were embedded together with HUVEC spheroids in fibrin gel in 24-well plates. The proportion of PANC-1 to HUVEC spheroids was 1:2. To control the potential effects of the spheroid co-culture, untreated/treated PANC-1 spheroids or HUVEC spheroids were embedded alone. Thereafter, the plates were incubated under hypoxic (1% O_2_) or standard culture conditions for 24 h. The spheroids were then fixed and their sprouts analyzed (see below). 

### 2.8. Western and Immunoblotting Analysis 

To better understand how molecular mechanisms via the different hyperthermia temperatures promote angiogenesis, we studied the ERK/MAPK pathway, which promotes cell proliferation, migration, and angiogenesis through the activation of transcription factors that increase VEGF transcription in tumor cells. We particularly paid attention to the proteins ERK44/ERK42 and their phosphorylated counterparts, p-ERK44/p-ERK42 and also HIF-1α. We used PANC-1 and BXPC-3 cell monolayers (controlled culture conditions, see above). In particular, after the pancreatic cancer cells were exposed to 43 or 47 °C for 1 h, cell lysis was performed using RIPA buffer [50 mM of TRIS-HCl pH 8.0, 150 mM of NaCl, 1% NP-40, 0.5% (*v*/*v*) Na-deoxycholate, 0.1% SDS (*w*/*v*), 1 mM of NaF, 1 mM of DTT, 0.4 mM of PMSF, 0.1 mM of Na_3_VO_4_, and protease inhibitor cocktail complete (Sigma-Aldrich GmbH, Steinheim, Germany)]. SDS polyacrylamide gels were used to separate the proteins and these were then blotted onto Immobilon^®^-P PVDF membranes (Merck KGaA, Darmstadt, Germany), as previously described [31]. Afterwards, the membranes were incubated with blocking buffer (5% (*w*/*v*) bovine serum albumin (BSA) or low-fat milk in Tris-buffered saline containing 0.1% (*v*/*v*) Tween20 (TBS-T)) for 1 h, and then rinsed and incubated overnight with rabbit antibodies against HIF-1α (Novus Biologicals, Abingdon, UK), p-ERK44_42, ERK44_42, VEGF (Invitrogen, Thermo Fisher Scientific, Waltham, MA, USA), GAPDH or β-actin (Cell Signaling, Frankfurt am Main, Germany) diluted at 1:800 to 1:5000 in blocking buffer. Horseradish peroxidase-conjugated goat anti-rabbit or goat anti-mouse secondary antibodies (both from Santa Cruz Biotechnology Inc., Santa Cruz, CA, USA) at a 1:5000 or 1:3000 dilution in blocking buffer, respectively, were used. Protein bands were detected using the chemiluminescence reaction method (EMD Millipore Corporation, Merck KGaA, Darmstadt, Germany). The ImageJ version 1.46r software (NIH, Bethesda, MD, USA) and Bio 1D version 15.08c software (Vilber Lourmat Deutschland GmbH, Eberhardzell, Germany) were used for the semi-quantitative analyses of protein bands. The protein expression was calculated relative to the respective protein loading control (GAPDH or β-actin).

### 2.9. Immunofluorescence Staining and Microscopy 

The intracellular immunofluorescence detection of HIF-1α was carried out on hyperthermia-exposed PANC-1 cells to assess the extent and localization of HIF-1α expression following hyperthermia. PANC-1 cell monolayers were seeded in pre-coated 8-well culture slides (BD-Biosciences Europe, Erembodegem, Belgium) and exposed to 43 °C or 47 °C hyperthermia for 1 h. Afterwards, the medium was changed and the cells were further cultured at standard conditions for 24 h. For immunofluorescence detection, cells were fixed with 3.7% (*v*/*v*) formaldehyde (Carl Roth GmbH, Karlsruhe, Germany) in culture medium, permeabilized for 5 min with 0.1% Triton-X100 (Sigma, Taufkirchen, Germany) in PBS and blocked with PBS containing 2% (*w*/*v*) BSA for 1 h. Phalloidin-Alexa Fluor^®^488 conjugate (Invitrogen) diluted at 1:150 in 0.5% BSA-supplemented PBS was used to stain the actin filaments. The cells were then incubated overnight at 4 °C with the anti-HIF-1α antibody (Novus, Germany, Cat No: NB100-479), diluted at 1:200 in PBS containing 0.5% BSA. Subsequently, cells were incubated in the dark for 30 min with the CY5-conjugated goat anti-rabbit IgG H&L secondary antibody (Abcam plc, Cambridge, UK) at a 1:200 dilution in PBS containing 0.5% BSA. Nuclear staining with Hoechst-33258, (AppliChem GmbH, Darmstadt, Germany) was carried out and the cells were mounted with Permafluor (Thermo-Fischer, Waltham, MA, USA), covered with glass coverslips and then subjected to confocal microscopy. The confocal laser scanning microscopy of the cells was performed on a Zeiss LSM 900 microscope (Zeiss, Jena, Germany) employing similar settings to those reported by Tansi et al. [32] using near-infrared fluorescent dyes in the wavelength range of Cy5, and also green fluorescent dyes.

### 2.10. Assessment of HUVECs Sprouting

The number of HUVEC sprouts as a measure of angiogenesis [33,34,35] was determined by counting individual sprouts per spheroid using the cellSens^TM^ image analysis version 1.3 software (Olympus, Tokyo, Japan). For each experimental condition, the number of sprouts per spheroid was determined as the mean value of ten spheroids.

### 2.11. Assessment of PANC-1 Spheroid Pseudopodia via Periodic Acid Schiff (PAS) and Hoechst stains of 3D Spheroids

The spheroid pseudopodia of tumor cells are considered to be a measure of their invasiveness into their surroundings [36,37,38,39]. To further characterize those pseudopodia, PANC-1 spheroids were stained for polysaccharides with the PAS for fungus staining kit (ScyTek Laboratories, Logan, UT, USA) according to the manufacturer’s instructions. The cell nuclei in embedded spheroids were stained with Hoechst 33258 (AppliChem GmbH, Darmstadt, Germany) at a 1:1000 dilution in HBSS. Spheroids were incubated in the dark for 10 min and then washed with HBSS and subjected to fluorescence microscopy to visually assess the presence or absence of nuclei and/or polysaccharides in the spheroid appendages. The number of PANC-1 pseudopodia per spheroid was determined as the mean value of ten spheroids.

### 2.12. Statistical Analyses

To verify the level of significance of the differences between the experiments, the graph pad prism 9 was used. Student’s *t*-test, one-way ANOVA and two-way ANOVA tests were implemented, when normality and equal variance were applicable. Each experiment was performed at least three times. Differences resulting in *p* < 0.05 were considered to be significant.

## 3. Results

### 3.1. Hyperthermia Treatment Exerts Cytotoxic Effects on PANC-1’s Viability and Proliferative Potential

**Figure 2:**
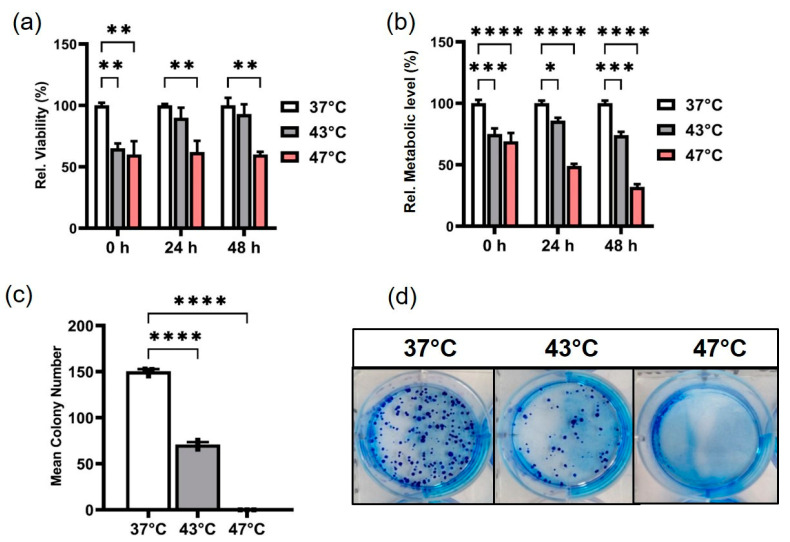
Strong hyperthermia (47 °C, 1 h) exerted the highest cytotoxic impact on PANC-1’s viability and proliferation. (**a**) Relative cell viability (CASY counter impedance spectroscopy) at 0, 24 and 48 h after strong/average hyperthermia; ** *p* < 0.01 for strong/average hyperthermia versus control at 37 °C. (**b**) Relative cellular metabolic activity (Alamarblue^®^ assay). **** *p* < 0.0001 for strong hyperthermia versus control at 37 °C. *** *p* < 0.001 for average hyperthermia versus control at 37 °C. * *p* < 0.05 for average hyperthermia (24 h) versus control at 37 °C (short-term effects). (**c**) Colony-forming ability at 15 days post-hyperthermia (Long-term effects). **** *p* < 0.0001 for strong/average hyperthermia versus control at 37 °C. (**d**) Pictures of colony plates for the different temperatures. Each bar represents the mean of *n* = 3 individual measurements and the standard deviation (SD) relative to the untreated controls grown at 37 °C.

Our data show that temperatures of 43 °C and 47 °C (average and strong) hyperthermia had varying degrees of cytotoxic effects on PANC-1 cells over time. Both hyperthermia temperatures resulted in a comparable decrease in viable cells immediately after hyperthermia compared to the untreated control (37 °C), according to the CASY counter, which measures cells with intact membranes (viable cells) (Figure 2a). Strong hyperthermia (47 °C, 1 h) caused a significant reduction in cell survival and recovery, and the early effects of the heat dose persisted even after 48 h (Figure 2a). On the other hand, cell recovery was observed in PANC-1 cells with average hyperthermia (43 °C, 1 h) after 24 h compared to 0 h. Most of the cells treated at 43 °C were still viable and had intact cell membranes 24 and 48 h after hyperthermia. Cell viability was nevertheless decreased with average hyperthermia (43 °C, 1 h) over time in comparison to the untreated control (Figure 2a). Similar to the cell viability assay, PANC-1’s metabolic activity and proliferation significantly decreased immediately after hyperthermia at both 43 °C and 47 °C compared to the 37 °C control (Figure 2b). In contrast to the control at 37 °C and average hyperthermia at 43 °C, 47 °C hyperthermia caused a more stable and significant decline in metabolic activity, and consequently, proliferation over time. Due to a significant loss of metabolic activity, cell proliferation was reduced by 50% and 30% (compared to the non-treated control, respectively) 24 and 48 h after the cells were subjected to 47 °C hyperthermia (Figure 2b). Even though the majority of the cells had intact membranes until 48 h after strong hyperthermia exposure (CASY measurement) (Figure 2a), the Alamarblue^®^ assay showed a less than 50% decrease in proliferation and metabolic activity in that same population after strong hyperthermia compared to the 37 °C control (set to 100%, Figure 2b). In addition, compared to the untreated control, strong hyperthermia (47 °C, 1 h) exhibited the highest long-term cytotoxic effects on PANC-1 cell proliferation, resulting in complete cell death in culture with no discernible colony after 15 days (Figure 2c,d). Average hyperthermia (43 °C) also exhibited a substantial long-term effect on PANC-1 proliferation, with a two-fold decrease in the PANC-1 mean colony number compared to the control cells at 37 °C (Figure 2c). Strong hyperthermia thus had the greatest short- and long-term cytotoxic effects on the proliferation and viability of PANC-1 cells. On the other hand, although average hyperthermia had a delayed cytotoxic effect on PANC-1, it still had a significant long-term effect on the disruption of cell viability and proliferation.

### 3.2. Hyperthermia Downregulated Intracellular HIF-1α Expression in PANC-1 Cells

**Figure 3:**
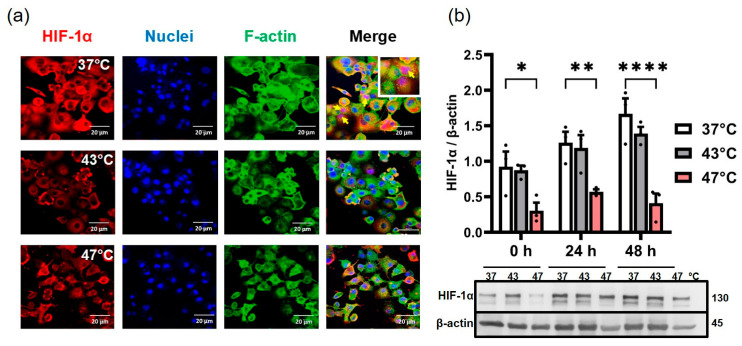
Hyperthermia downregulated HIF-1α expression in PANC-1 cells. (**a**) Representative confocal micrographs of HIF-1α immunostained PANC-1 cells 24 h post-hyperthermia. A characteristic decrease in the HIF-1α expression and nuclear accumulation (yellow arrows—nuclear accumulation of HIF-1α) is seen in the low-red-fluorescence intensity in hyperthermia-treated cells and substantiated by (**b**) Western blot analysis of HIF-1α expression. For the Western blot analysis, each bar represents the relative intensity of the protein bands compared to the respective protein loading controls. (* *p* < 0.05 for 47 °C hyperthermia versus control (37 °C) at 0 h, ** *p* < 0.01 for 47 °C hyperthermia versus 37 °C at 24 h, **** *p* < 0.0001 for 47 °C hyperthermia versus 37 °C, 48 h after therapy). Each bar represents the mean + SEM of *n* = 3 individual measurements.

Immunofluorescence detection showed a steady decrease in HIF-1α expression with hyperthermia treatment (Figure 3a) compared to the untreated control (37 °C). Similarly, HIF-1α nuclear accumulation (yellow arrow) was significantly reduced with hyperthermia. 

Western and immunoblotting analysis also showed a marked decrease in intracellular HIF-1α expressions after strong hyperthermia over time, compared to untreated controls (*p* < 0.01 & *p* < 0.0001; 24 h and 48 h, respectively, post-treatment) (Figure 3b). PANC-1 cells did not regain HIF-1α expression even 48 h after treatment. Average hyperthermia (43 °C, 1 h) had no significant effect on HIF-1α expression compared to untreated control (Figure 3b). Overall, hyperthermia downregulated HIF-1α expression and nuclear accumulation and did not induce intracellular hypoxia in PANC-1 cells.

### 3.3. Hyperthermia Alters the Secretion of Growth Factors in 2D PANC-1 Cells and the Ensuing Conditioned Media Does Not Drive Sprouting Angiogenesis in HUVEC Spheroids

**Figure 4:**
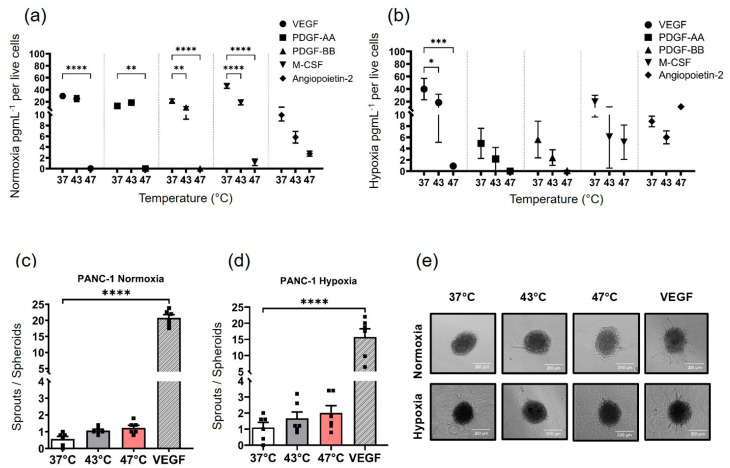
The secretion of growth factors is inhibited in hyperthermia-treated PANC-1 monolayer cells (2D culture condition, average or strong temperatures of 43 °C or 47 °C for 1 h). PANC-1 cells were subjected to different levels of hyperthermia and then cultured under standard or hypoxic (1% O_2_) conditions for 24 h. The culture media was then analyzed for the presence of angiogenic growth factors. Relative levels of growth factors secreted by hyperthermia-treated PANC-1 cells cultured under (**a**) normoxia and (**b**) hypoxia. **** *p* < 0.0001 for VEGF, PDGF-BB, M-CSF in strong/average hyperthermia versus control at 37 °C (normoxia). ** *p* < 0.01 for PDGF-AA and BB in strong/average hyperthermia versus control at 37 °C (normoxia). *** *p* < 0.001 and * *p* < 0.05 for VEGF in strong/average hyperthermia versus control at 37 °C (hypoxia). (**c**) Semi-quantitative analysis of sprout number of HUVEC spheroids under normoxia for 24 h and (**d**) hypoxia for 24 h in response to the conditioned medium from hyperthermia-treated PANC-1 cells (43 or 47 °C for 1 h, respectively) or non-treated ones (37 °C, normoxia, 24 h). (**e**) Representative light microscopy images of HUVEC spheroids treated with the conditioned medium. As a positive control for VEGF-mediated sprouting, HUVEC spheroids were also incubated in the presence of VEGF (10 ng/mL). The results are presented as mean ± SEM of *n* = 3 individual measurements, **** *p* < 0.0001 for VEGF-mediated sprouting (positive control) versus CM control (experimental control) at 37 °C. Scale bar: 200 µm.

Under normoxic conditions, strong hyperthermia (47 °C, 1 h) markedly reduced the secretion of VEGF, PDGF-AA, PDGF-BB, and M-CSF in PANC-1 cells 24 h after exposure compared to the control at 37 °C under normoxic conditions (Figure 4a). VEGF and PDGF-AA secretion were unaffected by average hyperthermia, but PDGF-BB and M-CSF secretion were noticeably down-regulated at 43 °C compared to the untreated control at 37 °C (Figure 4a). It is interesting to note that both hyperthermia temperatures prevented the hypoxia-induced secretion of proangiogenic factors. In comparison to untreated cells (37 °C) under hypoxia, both 43° and 47 °C hyperthermia significantly decreased VEGF secretion and suppressed hypoxia-induced growth factor secretion (Figure 4b). The conditioned medium obtained from PANC-1 cells subjected to hyperthermia treatment did not induce significant sprouting in HUVEC spheroids, even after incubation in normoxic or hypoxic conditions for 24 h. This was in comparison to the sprouting control (HUVEC spheroid treated with 10 ng/mL VEGF) (Figure 4c,d). A tendency of a temperature-dependent increase in sprouting activity was observed especially under hypoxia (Figure 4d,e). However, there was no significant difference between the hyperthermia-treated and the untreated control. Hence, hyperthermia significantly repressed the secretion of angiogenic growth factors (normoxia) and inhibited the hypoxia-induced secretion of growth factors in PANC-1 cells after 24 h. In addition, pro-angiogenic factors from hyperthermia-treated PANC-1 cells (43 °C) after 24 h do not singularly drive sprouting angiogenesis in HUVEC spheroids.

### 3.4. Hyperthermia-Treated PANC-1 3D Spheroids Stimulate the VEGF/MAPK/ERK Pathway, Which in Turn Drives Sprouting Activity in Endothelial Spheroids

**Figure 5:**
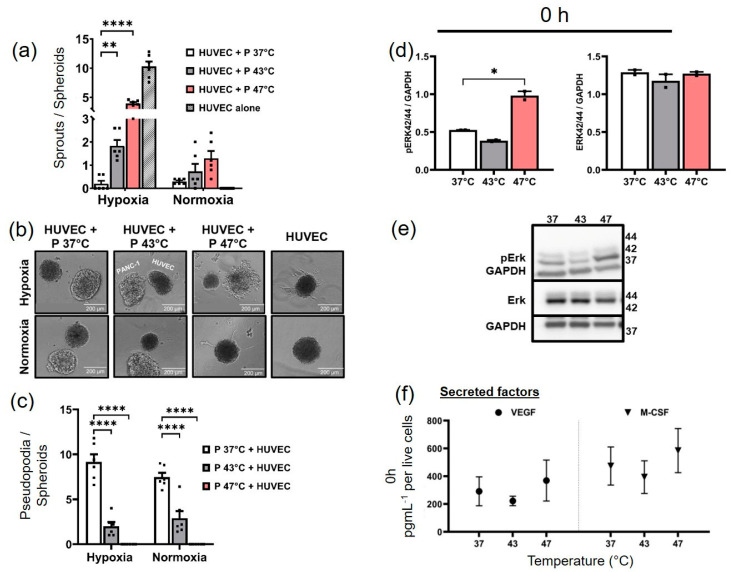
Co-culturing HUVEC spheroids with hyperthermia-treated PANC-1 spheroids may enable hypoxia-dependent endothelial sprouting in HUVEC spheroids via the VEGF/MAPK/ERK pathway. (**a**) Semi-quantitative analysis of sprout number of HUVEC spheroids when co-cultured with hyperthermia-treated PANC-1 spheroids (at 43 or 47 °C for 1 h) under hypoxic (1% O_2_) or normoxic conditions (standard culture conditions). **** *p* < 0.0001 or ** *p* < 0.01 for effect of hyperthermia-treated PANC-1 spheroids on HUVEC sprouts versus untreated PANC-1 spheroids. (**b**) Representative light micrograph of HUVEC spheroids co-cultured with PANC-1 spheroids. (**c**) Semi-quantitative analysis of the number of pseudopodia on PANC-1 spheroids embedded in fibrin matrix when co-cultured with HUVEC spheroids under hypoxic or normoxic conditions for 24 h. **** *p* < 0.0001 for PANC-1 sprout-like pseudopodia development in hyperthermia-treated versus untreated PANC-1 spheroids. (**d**) Western blot analysis of phosphorylated ERK1/2 (pERK 44/42) and ERK1/2 (ERK 44/42) expressions in PANC-1 cells immediately after hyperthermia (0 h). * *p* < 0.05 for strong hyperthermia versus control at 37 °C. (**e**) Blot images of (**d**). (**f**) Enhanced secretion of VEGF and M-CSF in PANC-1 cells immediately after strong hyperthermia (at 47 °C). The bars represent the mean ± SEM of *n* = 3 measurements. P = PANC-1.

A co-culture of 47 °C-treated PANC-1 spheroids with HUVEC spheroids promoted the hypoxia-induced sprouting of HUVEC spheroids more effectively than untreated (37 °C) PANC-1 spheroids (Figure 5a). There was a significant temperature-dependent increase in the sprouting activity of HUVEC spheroids under hypoxia conditions of 1% O_2_ (Figure 5a,b). Conversely, when PANC-1 spheroids were subjected to 43 °C treatment, they similarly allowed the sprouting of HUVEC spheroids under the hypoxia trigger, albeit to a lesser extent (1–2 sprouts per spheroid). Simultaneously, hyperthermia had a significant inhibitory effect on the invasiveness of PANC-1 within its surrounding fibrin matrix. Pseudopodia, or ‘sprout-like appendages’, were observed protruding from matrix-embedded PANC-1 spheroids and invading the matrix (Figure 5c). These pseudopodia are extensions of the PANC-1 spheroids that penetrate into the fibrin matrix but do not contain cells (negative nuclei stain); instead, they are polysaccharide-rich (positive PAS stain) extensions (Figure S2). However, as hyperthermia temperatures increased, the sprout-like invasion of the fibrin matrix by PANC-1 was substantially reduced (*p* < 0.0001), especially under strong hyperthermia conditions (47 °C, 1 h)compared to control (37 °C) (Figure 5c). Average hyperthermia also had a significant inhibitory effect (*p* < 0.0001) on PANC-1 invasiveness, reducing the number of sprout-like pseudopodia by approximately 4.5-fold under hypoxic conditions, compared to the untreated control. As such, strong and average hyperthermia were both found to inhibit PANC-1’s ability to invade the fibrin matrix, even under hypoxic conditions. 

In order to assess the impact of hyperthermia-treated PANC-1 spheroids on HUVEC spheroids, the immediate activation of the ERK/MAPK pathway in PANC-1 cells following hyperthermia was examined. This was necessary because the PANC-1 and HUVEC spheroids were co-cultured immediately after PANC-1’s exposure to hyperthermia. Remarkably, exposure to 47 °C hyperthermia treatment (0 h) significantly activated the ERK pathway, resulting in an increase in phosphorylated ERK compared to the control (*p* < 0.05) (Figure 5d,e). However, none of the hyperthermia temperatures had an effect on the total ERK expression at 0 h (Figure 5d,e). Further analysis revealed that strong hyperthermia induced the secretion of VEGF, M-CSF, and PDGF-BB in PANC-1 (0 h) (Figure 5f). After 24 h, no significant activation of ERK was observed in the hyperthermia-treated PANC-1 cells under normoxia or hypoxia. The total ERK expression remained unchanged for average and strong hyperthermia temperatures after 24 h (Figure S3). The 47 °C treatment inhibited intracellular VEGF expression compared to the untreated control (37 °C) 24 h post-hyperthermia (normoxia). However, under hypoxia stimulation, the PANC-1 cells treated at 47 °C regained their expression of intracellular VEGF. In summary, the hyperthermia treatment of PANC-1 spheroids may increase hypoxia-induced HUVEC sprouting via the VEGF/MAPK/ERK pathway. Nonetheless, while VEGF secretion was inhibited 24 h post-47 °C treatment, intracellular VEGF expression was not affected in hypoxic PANC-1. Hyperthermia at 43 °C did not affect intracellular VEGF expression under both normoxia and hypoxia conditions.

### 3.5. Similar Activation of the MAPK/ERK Pathway in BxPC-3 cells (2D) Due to Strong Hyperthermia Leads to the Initiation of Various Cellular Responses

**Figure 6:**
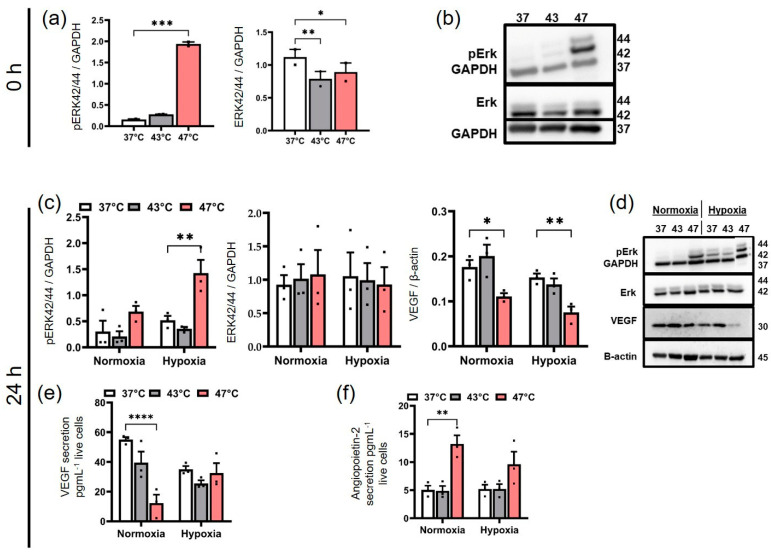
BxPC-3 cells respond to strong hyperthermia (47 °C for 1 h) with reduced de novo VEGF production, restored VEGF secretion, and the prolonged ERK activation of the pERK44/24 MAPKs pathway. (**a**) The expression levels of ERK1/2 (ERK 44/42) and phosphorylated ERK1/2 (pERK 44/42) in BxPC-3 cells immediately following hyperthermia (0 h, normoxia). *** *p* < 0.001 for strong hyperthermia versus control at 37 °C. * *p* < 0.05 for strong hyperthermia versus control at 37 °C. ** *p* < 0.01 for average hyperthermia versus control at 37 °C. (**b**) Representative blot images of (**a**). (**c**) Expression levels of pERK 44/42, ERK 44/42, and intracellular VEGF in BxPC-3 cells after hyperthermia and 24 h under normoxic or hypoxic conditions. ** *p* < 0.01 for strong hyperthermia versus control at 37 °C. * *p* < 0.05 for strong hyperthermia versus control at 37 °C; (**d**) Representative blot pictures of (**c**). At 24 h after hyperthermia, BxPC-3 cells released (**e**) secreted VEGF (**** *p* < 0.0001 for strong hyperthermia versus control at 37 °C) and (**f**) angiopoietin-2 into the culture medium under normoxia or hypoxia. ** *p* < 0.01 for strong hyperthermia versus control at 37 °C. Each bar in the western blot analysis depicts the relative level of protein expressions in relation to the respective protein loading controls (GAPDH and β-actin). Each bar represents the mean ± SEM of *n* = 3 individual measurements.

Similar to PANC-1 cells, immediate treatment with strong hyperthermia (47 °C, 1 h) significantly increased ERK phosphorylation in BxPC-3 cells compared to the untreated control (37 °C) (Figure 6a, b). However, unlike PANC-1 cells, BxPC-3 cells maintained ERK activation 24 h after strong hyperthermia treatment, especially under hypoxia (pERK44/42, Figure 6c). In contrast, average hyperthermia did not activate ERK immediately after treatment or 24 h later compared to the control at 37 °C (pERK44/42, Figure 6a–c). Interestingly, both hyperthermia temperatures distinctly reduced the total ERK expression in BxPC-3 cells immediately after therapy (Figure 6a, b). However, this effect was only transient, since 24 h later, the total ERK expression was comparable to the untreated cells at 37 °C. Interestingly, particularly strong hyperthermia (24 h post-therapy) also significantly decreased intracellular (de novo) VEGF expression (* *p* < 0.05) in BxPC-3 cells compared to the 37 °C control (Figure 6c). In contrast, the capacity for VEGF secretion was restored in hypoxic but not in normoxic BxPC-3 cells (both 24 h post-therapy, Figure 6e). The secretion of angiopoietin-2, a mediator of VEGF-dependent angiogenesis, was increased after the strong hyperthermia treatment of BxPC-3 cells (47 °C, 1 h) compared to the control (37 °C) when kept under normoxia and hypoxia (24 h post-therapy, Figure 6f). In contrast to strong hyperthermia, the average hyperthermia of BxPC-3 cells had no impact on the release of angiopoietin-2 compared to the control cells at 37 °C. Overall, the strong hyperthermia of BxPC-3 cells activates the MAPK/ERK pathway, reduces intracellular (de novo) VEGF expression while stimulating VEGF secretion, and increases angiopoetin-2 secretion under a hypoxic environment. 

### 3.6. HIF-1α Inhibition Down-Regulates Hyperthermia-Induced ERK Activation in Pancreatic Cancer Cells

**Figure 7:**
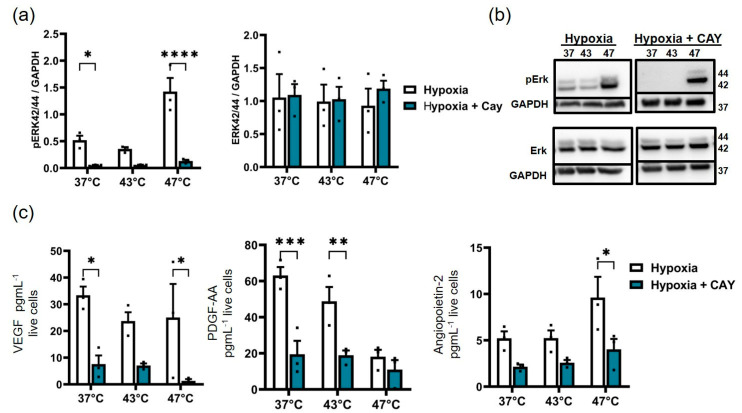
In BxPC-3 cells, the HIF-1α inhibitor CAY10585 reduces the secretion of growth factors and suppresses ERK1/2 phosphorylation. (**a**) Levels of pERK44/42 and ERK1/2 (ERK44/42) protein in BxPC-3 cells 24 h after hyperthermia (hypoxia or hypoxia + CAY10585). * *p* < 0.05 for hypoxia versus hypoxia + CAY10585 in the 37 °C control. **** *p* < 0.0001 for hypoxia versus hypoxia + CAY10585 in strong hyperthermia. (**b**) Representative blots of “b”. Each bar in the Western and immunoblot analysis depicts the relative density of protein bands in relation to the respective protein loading controls (GAPDH). (**c**) Decreased secretion of angiogenic growth factors from BxPC-3 cells into the culture medium in the inhibitor group (hypoxia + CAY10585) when compared to the hypoxia group. * *p* < 0.05 for hypoxia versus hypoxia + CAY10585 in (VEGF) 37 °C control and (VEGF, angiopoietin-2) strong hyperthermia 47 °C. ** *p* < 0.01 for hypoxia versus hypoxia + CAY10585 in (PDGF-AA) 43 °C and *** *p* < 0.001 in (PDGF-AA) 37 °C control. Each bar represents the mean ± SEM of *n* = 3 individual measurements.

We further studied the impact of HIF-1α inhibition on the downstream activation of the MAPK/ERK pathway in BxPC-3 cells, since those cells have shown a higher treatment susceptibility to hyperthermia than PANC-1 cells when held at post-therapeutic hypoxic conditions. To this end, 10 µM of the HIF-1α inhibitor CAY10585 (a small molecule inhibitor of HIF-1α accumulation and gene transcriptional activity) was applied to BxPC-3 cells (hyperthermia-treated or not) and incubated under hypoxic conditions (24 h). A HIF-1α-dependent presence of phosphorylated ERK was observed for average hyperthermia and non-treated controls, but it was HIF-1α-independent at higher treatment temperatures (47 °C; Figure 7a,b). HIF-1α inhibition, however, had no effect on the total ERK expression across all groups (Figure 7a,b). At the same time, HIF-1α inhibition resulted in a considerable down-regulation of proangiogenic factors secreted to the culture medium when cells were or were not treated with hyperthermia. The HIF-1α inhibition of strong hyperthermia-treated cells (47 °C, 1 h) specifically decreased VEGF secretion to the culture medium; this effect was comparable to that of non-hyperthermia-treated cells (37 °C, Figure 7c). Additionally, the inhibition of HIF-1α reduced the levels of PDGF-AA, particularly in average hyperthermia (43 °C, 1 h)-treated or non-treated (37 °C) cells. HIF-1α inhibition also reduced angiopoietin-2 secretion, particularly in 47 °C hyperthermia (Figure 7c). In contrast to BxPC-3, the treatment of native or hyperthermic-PANC-1 cells with the same HIF-1α inhibitor (10 µM) did not significantly reduce ERK phosphorylation, it had no impact on the overall expression of ERK, and it did not affect the secretion of growth factors by PANC-1 cells, including PDGF-AA, PDGF-BB, and M-CSF (Figure S3). Overall, HIF-1α inhibition had a higher impact on pERK expression and on the pro-angiogenic growth factor secretion of hyperthermia-treated BxPC-3 cells than on PANC-1 cells kept under hypoxia. 

## 4. Discussion

In this study, we observed that (a) hyperthermia alters the secretion of pro-angiogenic growth factors in pancreatic tumor cells and that (b) there is a shift in the angiogenic “cross-talk” between hyperthermia-treated tumor cells and endothelial cells; this interaction is dependent on ERK phosphorylation and the oxygen status of the tumor–endothelial cell environment after treatment. 

Our data on growth factor secretion by PANC-1 cells show that hyperthermia alters the secretion pattern of pro-angiogenic mediators. While strong hyperthermia (47 °C, 1 h) had a significant inhibitory effect on secretion, especially for VEGF and M-CSF-1, average hyperthermia did not cause any change in the VEGF secretion levels produced by PANC-1. Growth factor synthesis and secretion in pancreatic cells are affected by the steady decline in cell viability and metabolic function with hyperthermia therapy. As with other proteins, the complex and multi-step processes of transcription and translation are required to produce VEGF and PDGF [40], and these are indicative of efficient metabolic activity in tumor cells [41]. Therefore, the decreased growth factor secretion is a result of the decreased metabolic activity of PANC-1 cells after exposure to hyperthermia. This was supported by the long-term cytotoxic effects of hyperthermia on PANC-1 cells, which showed complete cell death after strong hyperthermia (47 °C, 1 h) and a significant reduction in cell recovery and survival after average temperatures (43 °C, 1 h), indicating a progressive cellular and metabolic “shut down” with hyperthermia therapy. 

In addition, hyperthermia significantly downregulated native HIF-1α expression and nuclear accumulation in PANC-1 cells. HIF-1α overexpression has been associated with the formation of metastatic tumors with a poor prognosis for patients with pancreatic cancer [42,43,44]. This is because HIF-1α is an important transcriptional factor that modulates the production of numerous key genes linked to tumor angiogenesis, including VEGF [45] and PDGF-BB [46]. HIF-1α upregulates VEGF production in pancreatic cancer cells by binding to the hypoxia response element of the VEGF-A promoter upon nuclear translocation [44,47]. Consequently, the severe inhibition of HIF-1α accumulation and nuclear translocation, particularly at strong hyperthermic temperatures (47 °C, 1 h), is linked to a decrease in growth factors produced following hyperthermia. Average hyperthermia did not significantly decrease HIF-1α expression in PANC-1 cells, but it inhibited its nuclear accumulation, which resulted in a considerable down-regulation of PDGF-BB and M-CSF 24 h after 43 °C hyperthermia (normoxia). Even though each protein has its own threshold of temperature stability, protein denaturation is one of the major physiologic effects of hyperthermia (42 to 47 °C), with nuclear proteins being particularly temperature-sensitive [48,49]. In light of this, the heat-induced denaturation of HIF-1α, particularly in the nucleus, may explain the unique repression of secreted growth factors even with a hypoxia trigger. 

The conditioned medium (CM) from the hyperthermia-treated PANC-1 cell monolayer (lateral cell-to-cell contacts only) did not promote significant sprouting angiogenesis in endothelial spheroids. Our data on the cross-talk between hyperthermia-treated PANC-1 monolayers and HUVEC spheroids indicate that the conditioned medium has almost no effect on endothelial cell sprouting under both normoxic and hypoxic conditions. This may be due to the decrease in metabolic function, HIF-1α accumulation and nuclear translocation [50] with hyperthermia treatment. Additionally, the untreated PANC-1 monolayers did not significantly promote sprouting. Studies by Erkan et al. [51] and Di Maggio et al. [52] show that pancreatic cancer cells, such as PANC-1, inhibit HUVEC growth and survival. One possible explanation for this is the increased expression of endostatin in pancreatic cancer cells such as PANC-1 [51]. Endostatin, an angiogenesis inhibitor, significantly inhibits endothelial growth and consequently angiogenesis [51,53]. This demonstrates that PANC-1 growth factors do not exclusively induce sprouting angiogenesis in endothelial spheroids, as the angiogenic balance is skewed towards antiangiogenic expressions in pancreatic cancer cells. Hence, our findings show that hyperthermia adversely affects the secretion of growth factors in pancreatic cancer cells even in the presence of hypoxia. This effect is attributed to the treatment’s potential to decrease (possibly by denaturation) the HIF-1α protein as well as impair the cellular and metabolic performance. 

The co-culture of hyperthermia-treated PANC-1 and HUVEC spheroids (self-organization of cells, intensive cell-to-cell contacts) revealed the temperature-dependent allowance of HUVEC sprouting compared to the co-culture of HUVEC with untreated PANC-1 spheroids. Compared to untreated PANC-1 spheroids, hyperthermic-PANC-1 spheroids (43 °C and 47 °C) significantly enabled hypoxia-induced endothelial sprouting. The increased cell-to-cell contact in the 3D PANC-1 spheroids, which enables increased cellular interaction similar to the in vivo situation, gives one secondary explanation to the sprouting differences observed between the hyperthermic 2D PANC-1 cells and 3D PANC-1 spheroids [54]. According to this study, another explanation for the increased sprouting activity may be related to strong hyperthermia’s immediate enhancement of phosphorylated ERK 1/2 in PANC-1 cells. This immediate response in ERK phosphorylation with hyperthermia is crucial because PANC-1 spheroids were co-cultured with HUVEC spheroids immediately after therapy. Several studies have shown that ERK/MAPK phosphorylation can activate the transcription of factors that promote VEGF production and angiogenesis [23,55,56]. In the present study, PANC-1 cells showed a simultaneous increase in VEGF secretion and ERK1/2 phosphorylation immediately after 47 °C hyperthermia. Similar to PANC-1 cells, the strong hyperthermia treatment of BxPC-3 cells resulted in distinct ERK1/2 phosphorylation compared to the untreated control immediately after therapy. However, in contrast to PANC-1 cells, ERK phosphorylation at 47 °C under hypoxic exposure persisted for 24 h after hyperthermia. One striking difference between the PANC-1 and BxPC-3 cell lines is the genetic mutation found in KRAS (Kirsten rat sarcoma viral oncogene homolog) [30]. While the PANC-1 cell line is thought to have oncogenic activated KRAS as a result of mutations, BxPC-3 is considered to have wild type (non-activated) KRAS [30,57]. Interestingly, the expression levels of KRAS have been related to ERK/MAPK phosphorylation, VEGF production and angiogenesis [58]. In agreement with this, we observed differences in the MAPK/ERK status of PANC-1 and BxPC-3 cells. Oncogenic ERK activation was present in PANC-1 cells and enhanced by 47 °C hyperthermia. In contrast, ERK1/2 was inactive in BxPC-3 cells and was phosphorylated only after 47 °C hyperthermia. The variations in HUVEC sprouting with temperature-enhanced ERK activation (47 °C and 43 °C PANC-1 spheroids) and native-oncogenic ERK activation (untreated PANC-1 spheroids) demonstrates that the alteration of ERK activation may strongly affect the angiogenic cross-communication between tumor cells and endothelial cells, although other pathways cannot be excluded. Additionally, the sustained activation of ERK1/2 under hypoxia in hyperthermic BxPC-3 and the increased sprouting activity under hypoxia in HUVEC spheroids co-cultured with hyperthermic PANC-1 spheroids support the involvement of hypoxia in increased ERK activation and nuclear translocation [59]. Under hypoxia, as ERK phosphorylation and nuclear translocation increase, activated ERK1 phosphorylates HIF-1α, further driving the transcription of the proangiogenic factors and gene expression that promote endothelial proliferation [23,59]. This explains the enhanced sprouting activity in the hyperthermic-PANC-1 co-culture under hypoxia. 

Endothelial cells with a high expression of VEGFR2 and VEGFR3 are known to compete and maintain tip cell positions during sprouting angiogenesis, as they are able to receive more VEGF [60]. In this study, VEGF secretion by pancreatic tumor cells (monolayer, non-treated) was 25 times lower than the concentration used to verify tip cell formation in HUVEC spheroids [12]; secondly, VEGF secretion was down-regulated after hyperthermia. Therefore, we postulate that the stimulation of the pancreatic tumor cells after hyperthermia on HUVEC spheroids was only sufficient for the formation of clearly differentiated tip cells due to the low concentration of VEGF post-therapy.

To confirm the role of HIF-1α in maintaining hyperthermia-induced ERK phosphorylation in BxPC-3 cells, we report the selective inhibition of ERK phosphorylation with HIF-1α inhibition in untreated and hyperthermia-treated BxPC-3 cells. This resulted in a decrease in secreted growth factors, VEGF, PDGF-AA, and angiopoietin-2. Although the HIF-1α inhibitor at a concentration of 10 µM greatly reduced the amount of HIF-1α accumulating in PANC-1 cells, it had no effect on phosphorylated ERK and the secretion of downstream growth factors. However, it is not entirely unexpected that the secretion of downstream growth factors occurred when HIF-1α was inhibited given the KRAS mutation in PANC-1 and the associated dysregulated signaling. 

**Figure 8:**
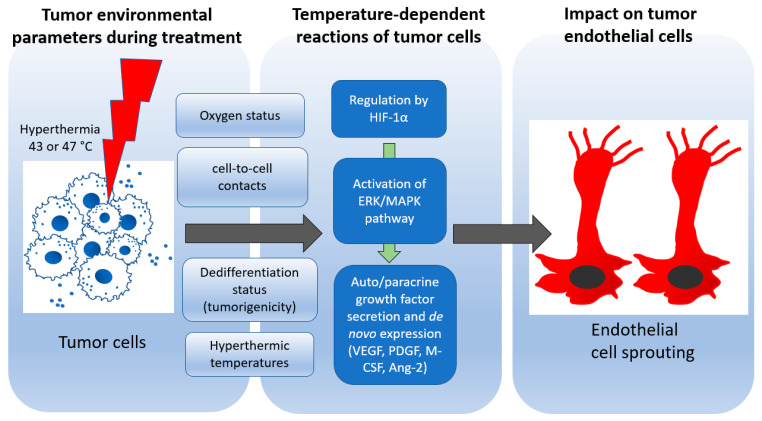
Identified players in the cross-talk of pancreatic adenocarcinoma cells and endothelial cells. Ang-2: Angiopoietin-2..

In PANC-1 cells, 47 °C hyperthermia decreased intracellular VEGF expression, which was restored under hypoxia. In contrast, secreted VEGF was reduced by 47 °C hyperthermia in both normoxia and hypoxia. In BxPC-3 cells, intracellular VEGF was also depleted by 47 °C hyperthermia with no recovery under hypoxia. However, secreted VEGF, especially at 24 h after 47 °C hyperthermia, was restored by hypoxia in BxPC-3 cells. This suggests that different processes contribute to the production of intracellular VEGF and secreted VEGF in the two pancreatic cancer cell lines. Nearly 90% of PDAC ductal epithelial cells have been observed to express intracellular VEGF, whereas neither the healthy pancreas nor chronic pancreatitis exhibit intracellular VEGF expression [61]. Numerous studies on PDAC show that VEGF acts directly inside cells as a signaling protein without being released [62,63,64], promoting endothelial migration and survival, hematopoietic stem cell survival, and osteoblast differentiation [62,63,64,65]. The malignant transformation of PDAC is associated with intracellular VEGF expression [61]. This has been attributed to the presence of mutant KRAS oncogene [66], which has been shown to initiate and upregulate intracellular VEGF expression in a mouse PDAC tumor model [67]. Similarly, the KRAS oncogene mutation in PANC-1 may contribute to the recovery of intracellular VEGF (depleted by hyperthermia) under hypoxia. The intracellular VEGF expression that was decreased by strong hyperthermia was not restored in BxPC-3 cells with wild-type KRAS, even in the presence of hypoxia. Notably, the hyperthermia treatment inhibited the formation of pseudopodia by PANC-1 spheroids (compared to non-treated ones), which has been postulated to be a sign of invasion and migration in the fibrin culture matrix [36,37,38]. In this context, average hyperthermia was sufficient to induce a significant decrease in PANC-1’s production of the polysaccharide-rich sprout-like pseudopodia in the fibrin matrix. Interestingly, hyperthermia not only inhibited hypoxia-induced invasiveness, but PANC-1 spheroids completely lost this invasive potential after strong hyperthermia. Similarly, it has been reported that high hyperthermia temperatures between 45 and 47 °C are more effective in inhibiting tumor invasion in a temperature-dependent manner than average hyperthermia temperatures (43 °C) [68,69]. Therefore, we expect that hyperthermia might well reduce the invasion and migration capacity of pancreatic tumor cells. Figure 8 gives a brief overview of the patterns identified in this study. 

It is of note that we used hot-air hyperthermia in our study, because it was technically well suited to our 2D and 3D culture systems. Nevertheless, there is some evidence to suggest that hot-air and the most frequently used hyperthermia technique in the clinical setting may have different effects on tumor cells. Therefore, further research activities should be conducted to understand the specific differences between these techniques.

## 5. Summary

In conclusion, our data show that the hyperthermic treatment of pancreatic adenocarcinoma affects endothelial cells, and this effect is primarily dependent on the oxygen status of the endothelial cell environment. Strong hyperthermia temperatures, shortly after treatment, activated ERK in both pancreatic cancer cell lines, whereas moderate hyperthermia temperatures did not activate the ERK/MAPK pathway. The angiogenic balance in PANC-1 was tipped by the hyperthermia treatment to favor sprouting activities in HUVEC spheroids while inhibiting invasion in PANC-1 spheroids. Overall, hyperthermia therapy of pancreatic tumor cells not only interferes with the known tumor functions, such as migration and invasion, but also affects the communication of tumor cells with surrounding endothelial cell nests. This may well be due to the interaction between the hyperthermia-activated MAPK/ERK pathway. In conclusion, the hyperthermia of pancreatic adenocarcinoma cells in vitro affects endothelial cells under defined environmental conditions (cell-to-cell contact, oxygen status, treatment temperature), with HIF-1α and VEGF secretion playing a role in a complex context. In pancreatic adenocarcinomas, which are known to be hypovascularized and to have limited therapeutic access, this complex effect of hyperthermia, if effectively exploited, could offer the therapeutic benefit of better drug delivery. 

## Data Availability

Data is available upon request.

## Supplementary Materials

The following are available online at https://www.mdpi.com/article/10.3390/biomedicines11082256/s1, Figure S1: Parameters during treatment of PANC-1 cells with hyperthermia, Figure S2: PANC-1 spheroids develop ‘sprout-like’ pseudopodia in the fibrin matrix., Figure S3: In PANC-1, strong hyperthermia (47 °C for 1 h) does not result in sustained ERK/MAPK activation 24 h later, Figure S4: The ERK1/2 phosphorylation and downstream growth factor secretions in PANC-1 cells were unaffected by the HIF-1α inhibitor CAY10585 at a concentration of 10 µM.
