# Supplementary material for: Hyperthermia Influences the Secretion Signature of Tumor Cells and Affects Endothelial Cell Sprouting
Source: Biomedicines. 2023 Aug 12;11(8):2256. doi: 10.3390/biomedicines11082256 (PMC10452125; doi:10.3390/biomedicines11082256)
Supplement: Supplementary file 1 [file biomedicines-11-02256-s001.zip › biomedicines-2504595-supplementary.pdf]

biomedicines

Article

Hyperthermia Influences the Secretion Signature of Tumor Cells and Affects Endothelial Cell Sprouting

Wisdom O. Maduabuchi [1], Felista L. Tansi [1], Regine Heller [2] and Ingrid Hilger [1,*]

[1] Department of Experimental Radiology, Institute of Diagnostic and Interventional Radiology, Jena University Hospital—Friedrich Schiller University Jena, Am Klinikum 1, D-07747 Jena, Germany; wisdom.maduabuchi@med.uni-jena.de (W.O.M.); felista.tansi@med.uni-jena.de (F.L.T.).

[2] Institute of Molecular Cell Biology Center for Molecular Biomedicine (CMB), Hans-Knöll-Str. 2, D-07745 Jena, Germany; regine.heller@med.uni-jena.de

* Correspondence: ingrid.hilger@med.uni-jena.de; Tel.: +49-3641-9325921

Supplementary Data S1: Parameters during treatment of PANC-1 cells with hyperthermia

PANC-1 cells / Spheroids were treated with hyperthermia at 47°C or 43°C for 1 h as described in the method section of the article. The cumulative equivalent minute (CEM43) is a measure of the effect of the temperature exposure to cell death; the thermal dose.

A

Hyperthermia	43°C	47°C
Treatment temp. (°C)	43 ± 0.2	47 ± 0.0
CEM43 (min)	65 ± 8.5	1003 ± 44
Treatment time (min)	61 ± 0.5	60 ± 0.5

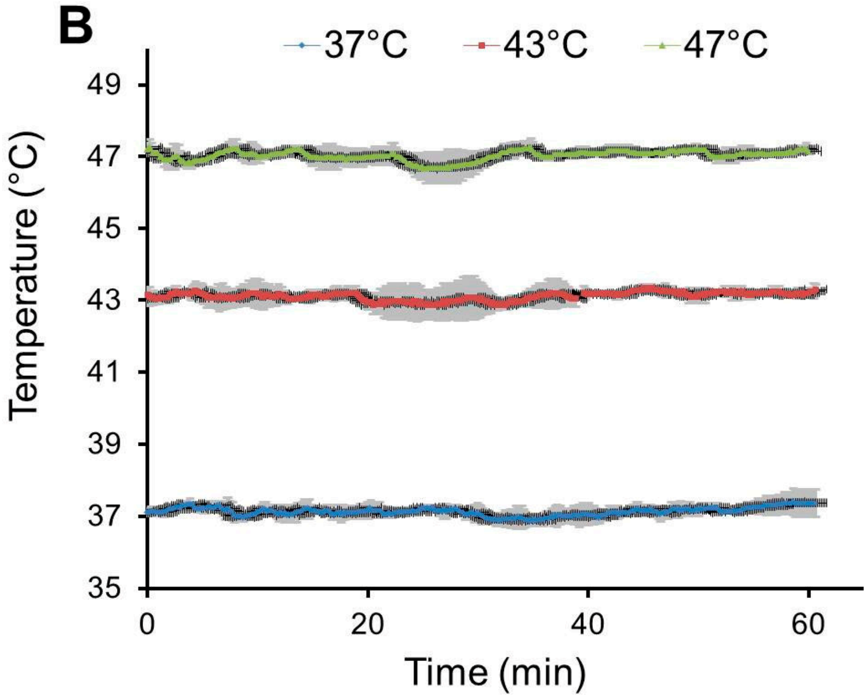

Figure S1: Parameters during treatment of PANC-1 cells with hyperthermia . CEM: cumulative equivalent minutes. **A)** Treatment parameters, **B)** Representative temperature curves recorded during treatment, grey shade represents the standard deviation.

Supplementary Data S2: PANC-1 spheroids develop 'sprout-like' pseudopodia in the fibrin matrix.

PANC-1 spheroids produce sprout-like extensions (pseudopodia) that infiltrate the fibrin matrix when placed in the matrix, either alone or in co-culture. We demonstrate that these pseudopodia, which have been used as a measure of invasiveness in PANC-1, are not made up of cells (negative nuclei stain). However, they are polysaccharide rich extensions that are derived from the outer cells lining the spheroids.

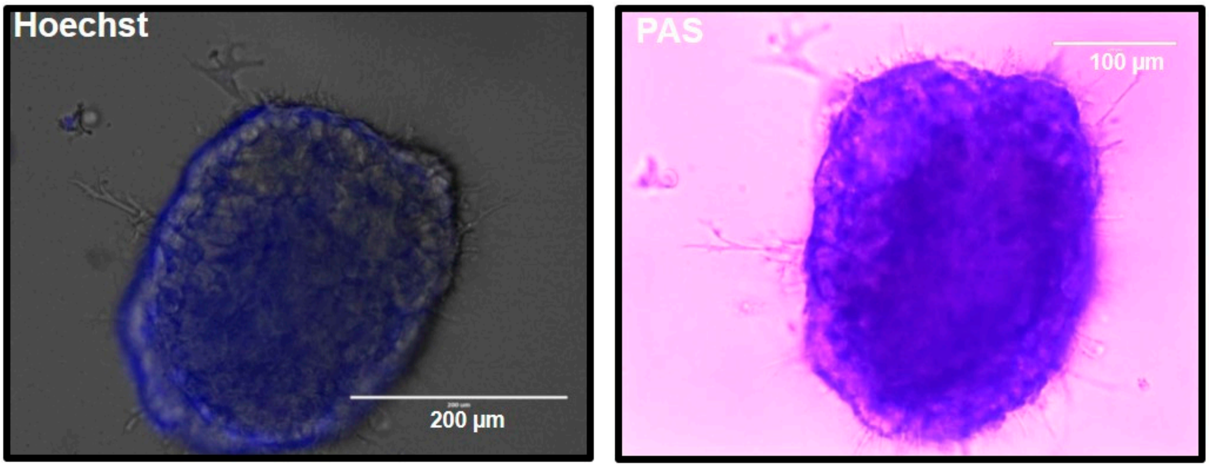

Figure S2: Representative fluorescence (for Hoechst, Scale bar 200 µm) and light (for PAS, Scale bar 100 µm) micrographs of a PANC-1 spheroids showing the penetration of polysacchride-rich pseudopodia into the fibrin matrix.

Supplementary Data S3: In PANC-1, strong hyperthermia (47°C for 1 h) does not result in sustained ERK/MAPK activation 24 h later.

24 h following strong hyperthermia, total ERK expression and phosphorylated ERK expression in the hyperthermia group are comparable to the untreated control at 37°C. Intracellular VEGF expression was adversely affected by strong hyperthermia in PANC-1. After 24 h, intracellular VEGF expression was restored in PANC-1 cells post incubation in hypoxia, whereas PANC-1 cells further incubated under standard conditions did not regain expression of intracellular VEGF.

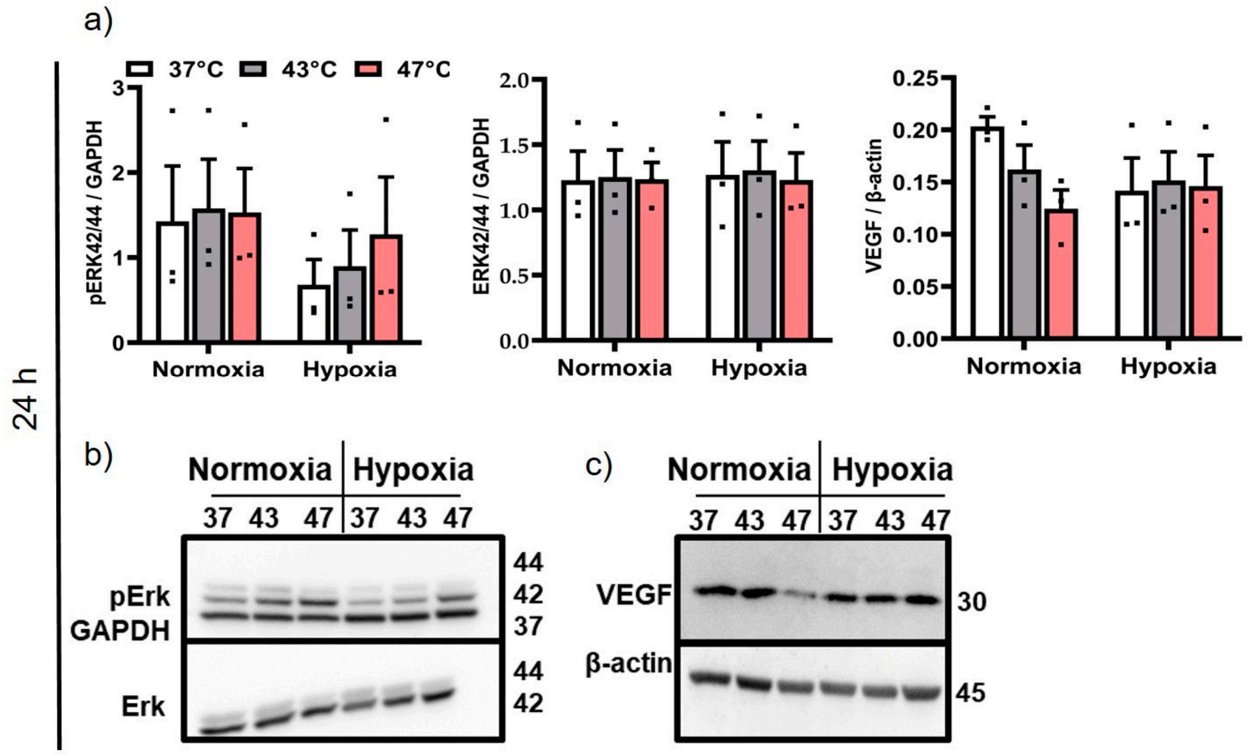

Figure S3: Activation of the ERK/MAPKs pathway in PANC-1 is not sustained 24 h after strong hyperthermia (47°C for 1 h). A) Western blot analysis of pERK44/42, ERK44/42 & intracellular VEGF expressions 24 h after hyperthermia under normoxia or hypoxia in PANC-1 cells. **B)** Representative blot images of A. For the western blot analysis, each bar represents the relative level of the protein expressions as compared to the respective loading controls. Each bar represents the mean±SEM of n=3 individual measurements.

Supplementary Data S4: The ERK1/2 phosphorylation and downstream growth factor secretions in PANC-1 cells were unaffected by the HIF-1α inhibitor CAY10585 at a concentration of 10 µM.

HIF-1α inhibitor CAY10585 at a concentration of 10 µM had no discernible effect on the ERK phosphorylation of native or hyperthermic-PANC-1 cells. Additionally, PDGF-AA, PDGF-BB, and M-CSF production by PANC-1 cells was unaffected by the inhibition of HIF-1α (10 µM).

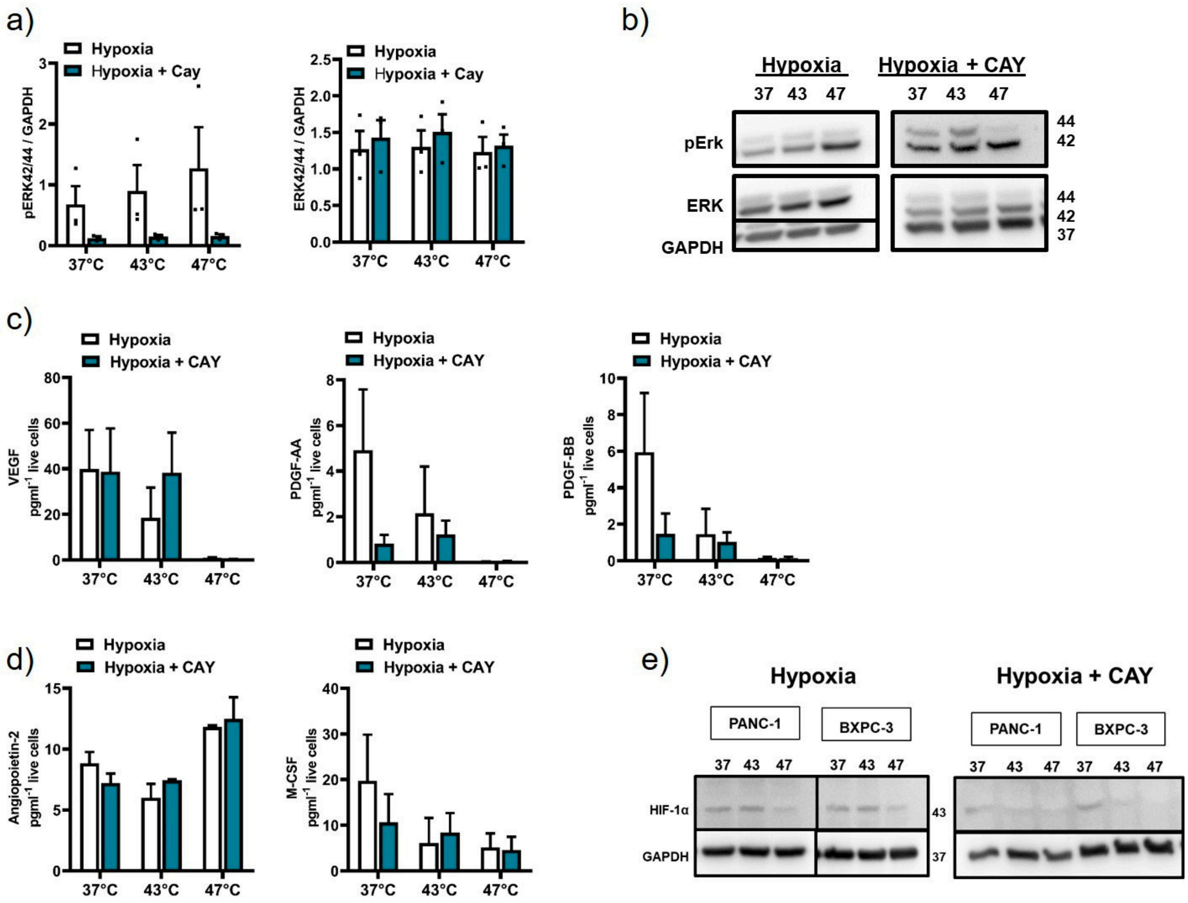

Figure S4: The HIF-1 inhibitor CAY10585, at a concentration of 10 μM, had no significant effect on ERK1/2 phosphorylation or secretions of downstream growth factors in PANC-1 cells. A) Levels of phosphorylated ERK1/2 (pERK 44/42) and ERK1/2 (ERK 44/42) in PANC-1 cells 24 h after hyperthermia (hypoxia or hypoxia + CAY10585). B) Representative blot images of A. Relative levels of angiogenic growth factors C) VEGF, PDGF-AA, PDGF-BB D) Angiopoietin-2 and M-CSF secreted from PANC-1 cells into the culture medium. E) Degraded HIF-1α protein detected at 43 kDa in both PANC-1 and BxPC-3 cells. HIF-1α inhibitor (CAY10585) inhibits HIF-1α accumulation, no detection of HIF-1α protein observed. Each bar represents the mean ± SEM of n=3 individual measurements. For the Western blot analysis, each bar represents the relative level of the protein expressions as compared to the respective protein loading controls.
